# Signal-enhanced real-time magnetic resonance of enzymatic reactions at millitesla fields†

**DOI:** 10.1039/d0sc04884d

**Published:** 2020-10-30

**Authors:** Sergey Korchak, Anil P. Jagtap, Stefan Glöggler

**Affiliations:** NMR Signal Enhancement Group, Max-Planck-Insitute for Biophysical Chemistry Am Faßberg 11 37077 Göttingen Germany stefan.gloeggler@mpibpc.mpg.de; Center for Biostructural Imaging of Neurodegeneration of UMG Von-Siebold-Str. 3A 37075 Göttingen Germany

## Abstract

The phenomenon of nuclear magnetic resonance (NMR) is widely applied in biomedical and biological science to study structures and dynamics of proteins and their reactions. Despite its impact, NMR is an inherently insensitive phenomenon and has driven the field to construct spectrometers with increasingly higher magnetic fields leading to more detection sensitivity. Here, we are demonstrating that enzymatic reactions can be followed in real-time at millitesla fields, three orders of magnitude lower than the field of state-of-the-art NMR spectrometers. This requires signal-enhancing samples *via* hyperpolarization. Within seconds, we have enhanced the signals of 2-^13^C-pyruvate, an important metabolite to probe cancer metabolism, in 22 mM concentrations (up to 10.1% ± 0.1% polarization) and show that such a large signal allows for the real-time detection of enzymatic conversion of pyruvate to lactate at 24 mT. This development paves the pathways for biological studies in portable and affordable NMR systems with a potential for medical diagnostics.

## Abstract 2

We demonstrate that metabolism can be monitored in real-time with magnetic resonance at milli-tesla fields that are 1000 fold lower than state-of-the-art high field spectrometers.

## Graphical abstract

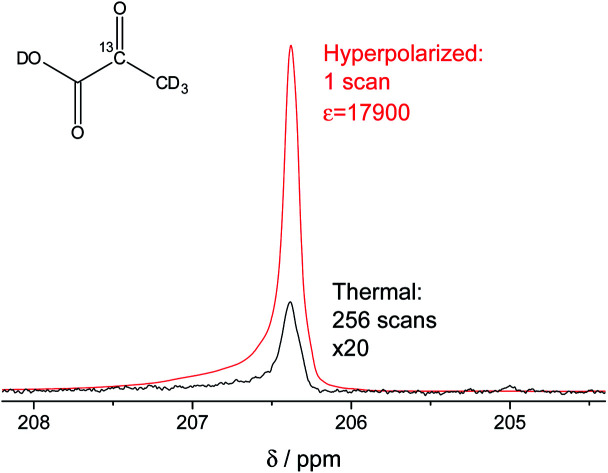

## Introduction

Nuclear magnetic resonance (NMR) is a versatile technique routinely applied in chemical analysis, structural biology and for medical diagnosis.^1–3^ Due to small energy differences of nuclear spin populations only a few spins per million contribute to the observable NMR signal even in state-of-the-art superconducting high-field magnets.^3^ Overpopulating one specific spin state beyond thermal equilibrium limitations allows enhancing NMR signals by several orders of magnitude. This process is referred to as hyperpolarization.^4–28^ While hyperpolarization has gained a lot of attention in the past decades, especially in biological sciences and in combination with high-field NMR and magnetic resonance imaging (MRI), it also offers potential for technological advances at very low to zero magnetic fields and not only for typical Faraday detectors.^29–31^ It especially offers potential for more sensitive magnetic field sensor technologies that have matured in recent years and include: atomic magnetometers (AM),^32–36^ superconducting quantum interferences devices (SQUIDs)^37–39^ and nitrogen vacancy (NV) diamond magnetometers.^40–47^ All of these sensors operate best at very low magnetic fields ranging from several millitesla (SQUID and NV magnetometers) to zero-field (AM), the so-called zero and ultra-low field regime (ZULF) starting on the order of 10 millitesla. It was shown, that NMR spectroscopy can be performed with all of the described sensors in the ZULF regime.^32,33,37,41^ In order to obtain sufficient signal, prepolarization at high magnetic fields or long averaging times are necessary. Additionally, some examples were demonstrated in which hyperpolarization enhances signals that can be rapidly detected at low magnetic fields.^29–31,33,39^ Although the hyperpolarized metabolites were Faraday detected at as low as 47 mT field and even *in vivo*,^29^ no enzymatic conversion in millitesla fields resolving different metabolites and desired kinetic parameters was demonstrated. Proof of principle experiments to monitor enzymatic reactions would pave pathways for efficient, low-cost and portable analytical and medical devices with the potential to detect the metabolism of single cells in real-time *via e.g.* NV-diamond sensors.

In this article, we demonstrate that the enzymatic conversion of hyperpolarized 2-^13^C-pyruvate into 2-^13^C-lactate can be monitored in real-time at millitesla fields. Pyruvate is an important metabolite and is currently investigated in clinical hyperpolarization studies as a probe for cancer.^28^ Even at such low magnetic fields, we obtain high-resolution NMR spectra containing information of different chemical moieties *via* the chemical shift parameter and the electron-mediated spin–spin (*J*-) coupling. Based on the highly resolved real-time NMR experiments we are able to devise kinetic parameters of the enzymatic pyruvate–lactate conversion in a portable low field (millitesla) NMR system without the requirement of a dedicated high-field magnet.

**Fig. 1:**
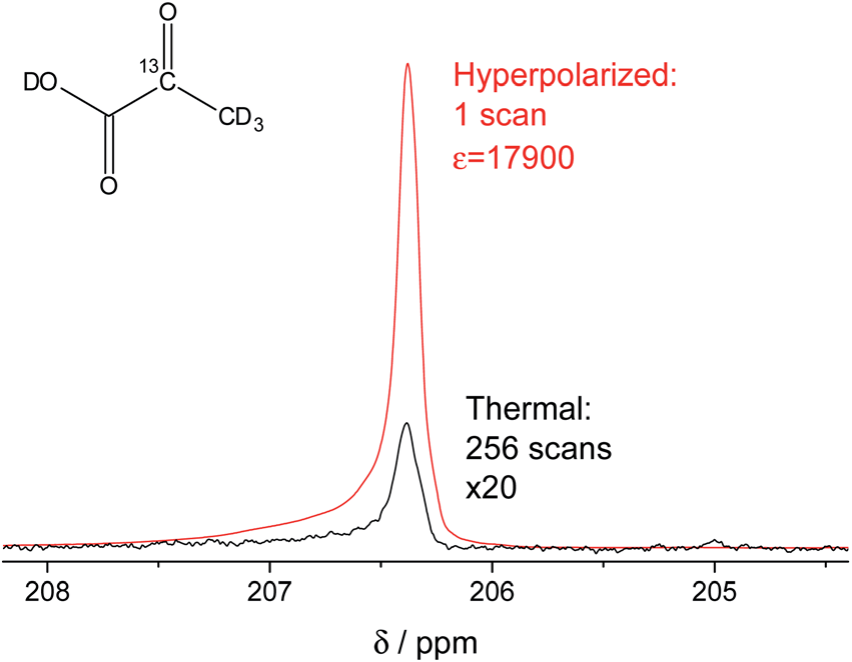
Signal enhanced 2-^13^C-pyruvate-d_3_. Red: ^13^C-NMR spectrum of hyperpolarized free 2-^13^C-pyruvate-d_3_ detected at *B*_0_ = 7 T and 320 K in a single scan with a 90° pulse after hydrolysis of the hydrogenation product using a carbonate solution (pH ∼ 10). Black: Thermally polarized ^13^C-NMR spectrum of the same sample and at the same conditions with 256 scans, 300 s repetition time and 20-fold enlarged. The signal enhancement corresponds to 17 900-fold at the given field. The hyperpolarization here corresponds to 10.2% with 10.1% on average over 3 experiments. Only a minor amount of side products are seen after hydrolysis (see also Fig. S5†).

In our study, we have accomplished to hyperpolarize 2-^13^C-pyruvate-d_3_ (Fig. 1) within seconds using *para*-hydrogen induced polarization (PHIP)^9,10,13^ to 10.1% ± 0.1% in 22 mM concentrations. PHIP relies on the conversion of singlet spin order of *para*-hydrogen into observable magnetization.^17^ This can be performed *via* a pairwise hydrogen addition to unsaturated bonds. As unsaturated precursors are not available for many relevant metabolites, the concept of PHIP-SAH (PHIP by means of sidearm hydrogenation) was introduced.^13^ It requires to combine a metabolite of interest and an unsaturated side chain as a chemical precursor. The side chain can be hydrogenated with *para*-hydrogen and the spin order subsequently transferred to a nucleus of interest within the metabolite. Rapid chemical cleavage of the side chain releases the metabolite for biological investigations. Here, we have achieved the efficient hyperpolarization of 2-^13^C-pyruvate-d_3_ with *para*-hydrogen by combining a rational side chain design and polarization transfer *via* the ESOTHERIC experiment (efficient spin order transfer to heteronuclei *via* relayed INEPT chains).^19,20^ Details on the procedure are described in the ESI.† One feature to achieve high levels of polarization was to deuterate the pyruvate to remove additional ^1^H–^13^C-couplings, which may affect the pulsed transfer experiment. The transfer was conducted in a commercial spectrometer at 7 T.^19^ The hyperpolarization results of 22 mM 2-^13^C-pyruvate-d_3_ are depicted in Fig. 1. Overall we achieve an average polarization of 10.1% which corresponds to a signal enhancement of about 17 900-fold compared to the thermal spectrum at a magnetic field of *B*_0_ = 7 T or about 5 million enhancement factor at 24 mT.

## Results and discussion

**Fig. 2:**
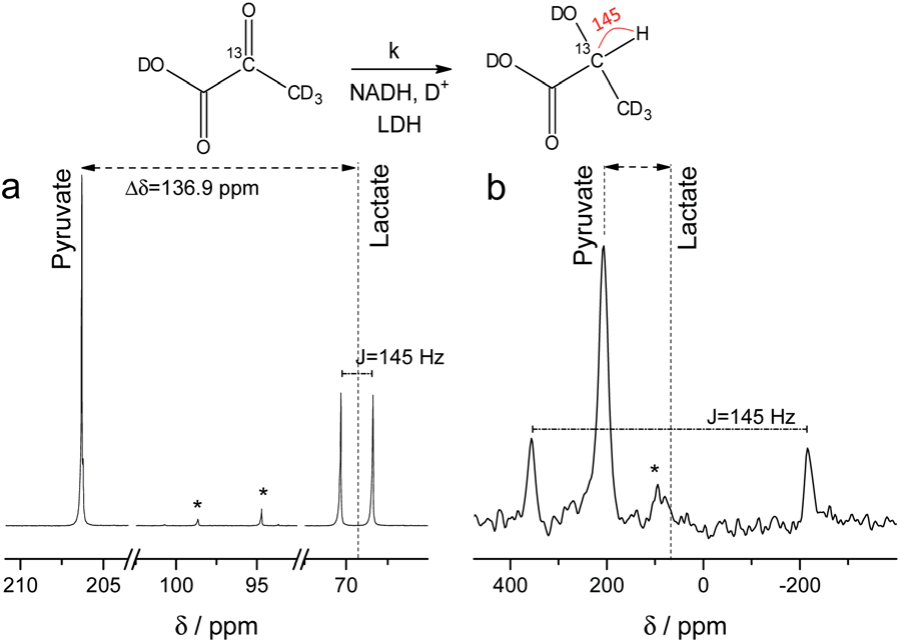
^13^C spectra of hyperpolarized 2-^13^C-pyruvate-d_3_ and 2-^13^C-lactate-d_3_ at high and low field. Spectra acquired with a single scan during the enzymatic pyruvate-to-lactate-conversion with lactate dehydrogenase at (a) *B*_0_ = 7 T and (b) *B*_0_ = 24 mT. The pyruvate is converted into lactate leading to a change in chemical shift of the ^13^C spins. Additionally, a large *J*-coupling of 145 Hz becomes observable as one proton is introduced into the otherwise deuterated lactate. * denotes hydrated form of pyruvate and side products of cleavage that do not participate in the conversion.

NMR setups working at millitesla magnetic fields experience lower chemical shift resolution, because the chemical shift is linear dependent on the magnetic field. However, the spin–spin interaction is independent of the magnetic field and in particular heteronuclear *J*-couplings such as the ^1^H–^13^C-coupling can be resolved even at zero-field to perform high-resolution NMR.^48^ We are exploring this fact here to distinguish between 2-^13^C-pyruvate-d_3_ and 2-^13^C-lactate-d_3_. Since the perdeuterated pyruvate does not contain any ^1^H–^13^C couplings we expect one major peak that is slightly broadened by the three deuterium interacting with the ^13^C spin. When 2-^13^C-pyruvate is enzymatically converted into 2-^13^C-lactate in the presence of protonated nicotinamide adenine dinucleotide (NADH), one proton is introduced into the molecule leading to a dominant ^1^H–^13^C coupling of 145 Hz. The difference in *J*-couplings of these pyruvate and lactate can then be used to distinguish them even without chemical shift difference. We have explored this pyruvate–lactate conversion starting from hyperpolarized 2-^13^C-pyruvate-d_3_ and the results for experiments at high-field (7 T) and low-field (24 mT) are depicted in Fig. 2. Hyperpolarized 2-^13^C-pyruvate-d_3_ is transformed into 2-^13^C-lactate-d_3_ under the action of lactate dehydrogenase (LDH) in presence of the reduced form of nicotinamide adenine dinucleotide (NADH). At high field (Fig. 2a), the ^13^C species of 2-^13^C-pyruvate-d_3_ and 2-^13^C-lactate-d_3_ are well separated by a large chemical shift difference of 136.9 ppm which corresponds to a frequency difference of about 10 kHz (*B*_0_ = 7 T). In addition, the large heteronuclear ^1^H–^13^C-coupling is observed. At low field (Fig. 2b), the large heteronuclear *J*-coupling of lactate allows to distinguish it from the pyruvate signal. We note that we achieve a linewidth of 4 Hz for ^13^C in our low field setup which is a limitation given by the stability of the applied current source and residual couplings to the deuterons. The homogeneity hence amounts to 15 ppm (with residual ^2^H–^13^C *J*-coupling broadening) over the sample volume. Due to the large chemical shift difference of 136.9 ppm and a field homogeneity of 15 ppm, we are even able to resolve the chemical shift difference between lactate and pyruvate. At 24 mT, the frequency difference amounts to 27.4 Hz which is well above the linewidth and hence resolvable as indicated in Fig. 2b. Additionally, the observable chemical shift difference allows to distinguish the desired peaks from minor cleavage side-products and the hydrated form of pyruvate. Overall, the hyperpolarized spectra at 24 mT yield the same information in single scan experiments as in the high magnetic field.

**Fig. 3:**
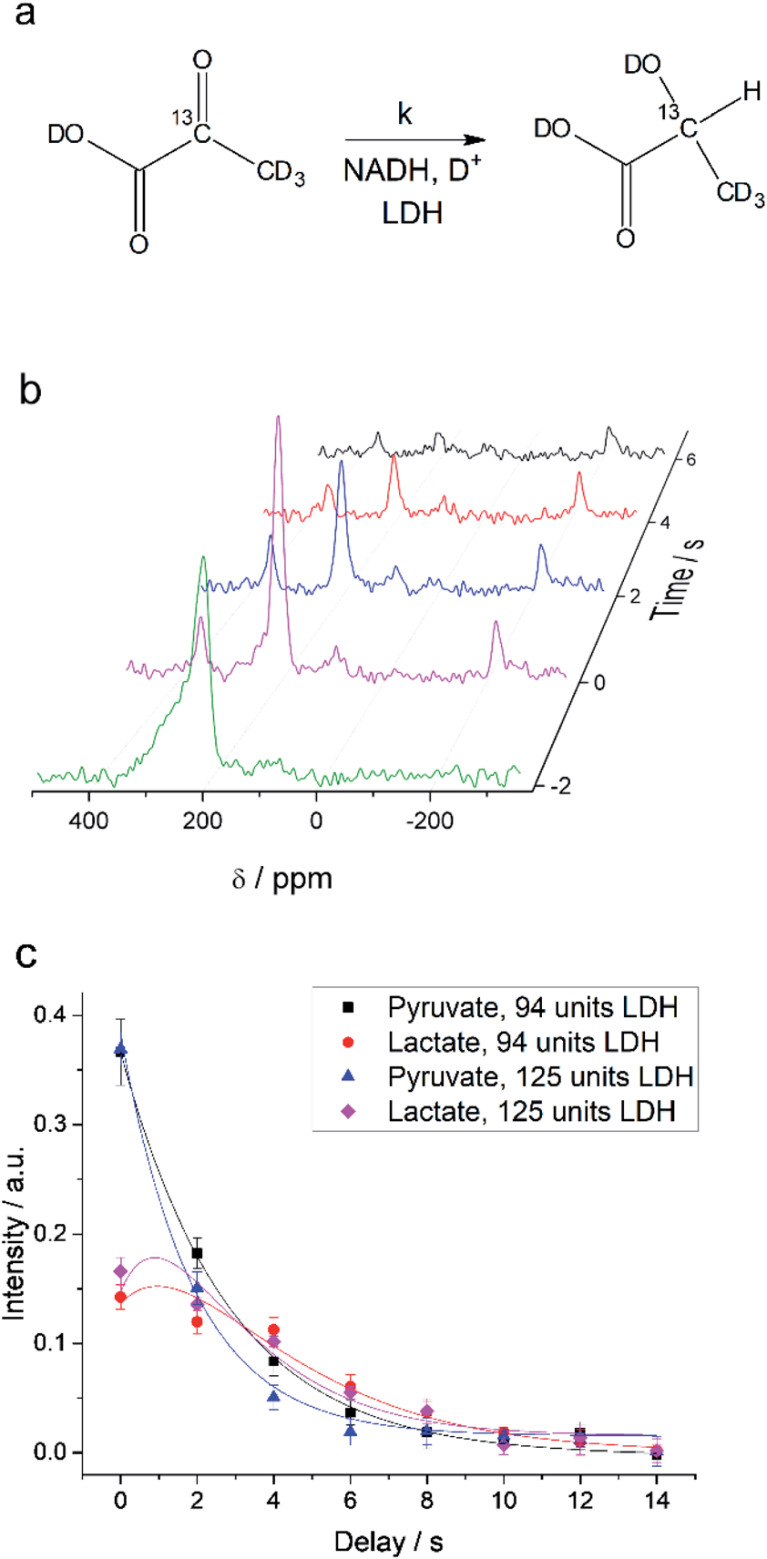
Enzymatic real-time dynamics at low magnetic field. (a) Schematic of the reaction from 2-^13^C-pyruvate-d_3_ to 2-^13^C-lactate-d_3_ with NADH and in the presence of LDH. (b) Single scan hyperpolarized spectra at 24 mT acquired in steps of two seconds show the conversion. Initially only 2-^13^C-pyruvate-d_3_ is observed (detected after a 6° pulse) that is consecutively converted into lactate (detected with 22° pulses). Shims are optimized for a larger sample after addition of the LDH solution. (c) Kinetics plots as an average of three experiments (error bars give the standard deviation) of the conversion of 2-^13^C-pyruvate-d_3_ to 2-^13^C-lactate-d_3_ together with simulated curves of the applied model. The reaction is characterized by the unimolecular rate constant *k*. To start the reaction, LDH, 20 mM NADH in 0.3 ml 20% HEPES buffer (1 M) in D_2_O was added to hyperpolarized pyruvate (22 mM) inside the low-field setup at 298 K. Zero time is set to the end of the mixing of the solution. According to the model described in the main text, the lactate relaxation rate constants are *R*^Lac^_1_ = 0.47 ± 0.03 s^−1^ and 0.50 ± 0.03 s^−1^ and the reaction rate constants are *k* = 0.31 ± 0.03 s^−1^ and 0.39 ± 0.04 s^−1^ for 94 units and 125 units of LDH correspondingly. *R*^Pyr^_1_ = 0.023 s^−1^ was measured in a separate experiment and set as a fixed parameter.

Interconversion of hyperpolarized pyruvate and lactate is currently investigated in hyperpolarized cancer studies as sensor for elevated metabolism.^28^ This metabolic step is promoted by lactate dehydrogenase and is schematically depicted in Fig. 3a. At 24 mT we are able to follow this conversion in real-time (Fig. 3b) due to the possibility of distinguishing 2-^13^C-pyruvate and 2-^13^C-lactate *via* the heteronuclear *J*-coupling and chemical shift difference. In Fig. 3c we show averaged decay curves of 2-^13^C-pyruvate-d_3_ and build-up of 2-^13^C-lactate-d_3_ derived from low-field experiments. The real-time dynamics for each repetition are shown in the ESI.† In Fig. 3b, we have acquired a signal of the initial 2-^13^C-pyruvate-d_3_ with a low flip angle pulse and before addition of the enzyme. After injection of the enzyme the reaction starts and the lactate signals appear. We probe the reaction in increments of two seconds following small flip angles leaving the most magnetization intact, thus allowing for deriving a parameter to evaluate the reaction kinetics (Fig. 3c).

Evaluating the kinetics of pyruvate to lactate have been a challenging task in pure enzymatic reactions with LDH over the past decades.^49^ Fortunately, no substrate inhibition under our conditions were operative because the pyruvate is freshly generated by hydrolysis and has no time to form a ternary complex with NAD+ and LDH during 16 s of the overall enzymatic reaction time.^50^ At low concentrations, first order reaction takes place according to the Michaelis–Menten equation. Due to the so obtained concentration dependence of pyruvate we have investigated whether a first order derived kinetic model is suitable to extract relevant reaction parameters of the reaction.^51^ We would like to note that the obtained apparent reaction rate constant will only serve as a parameter to differentiate between LDH concentrations. Assuming excess of NADH and first order kinetics with respect to the pyruvate concentration, the reaction catalyzed by LDH is the following:1
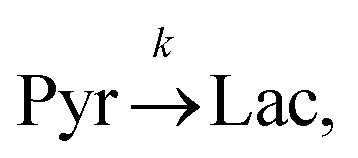
where *k* is the pyruvate–lactate conversion rate constant that depends on LDH concentration. The reaction is initiated by mixing NADH and LDH with hyperpolarized pyruvate in a solution. In general the reaction is an exchange but because pyruvate is our initial compound and the equilibrium is strongly shifted to the lactate side, we consider only the conversion of pyruvate to lactate. Thus, the magnetization of the conversion can be described by two differential equations:2
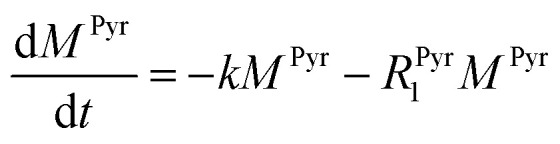
3
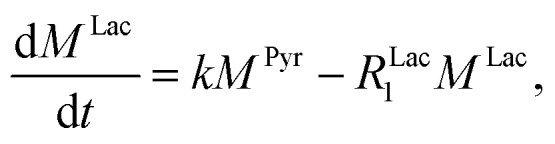
where *M*^Pyr^, and *M*^Lac^ are the pyruvate and lactate magnetizations and *R*^Pyr^_1_, and *R*^Lac^_1_ are the longitudinal relaxation rate constants of the magnetization, correspondently. In these equations the *R*^Pyr^_1_ is known. The relaxation rate constant of the 2-^13^C spin in 2-^13^C-pyruvate-d_3_ was determined prior to the conducted kinetics experiment at 24 mT with hyperpolarized samples (Fig. S7†) and amounts to *R*^Pyr^_1_ = 0.023 s^−1^. In order to obtain the relaxation rate for 2-^13^C-lactate-d_3_ and the conversion rate, we have to make further considerations with respect to the hyperpolarized signals. For the detection of the magnetization, a radio frequency pulse angle 22° well below 90° was used to allow for consecutive measurements and to not destroy the hyperpolarized magnetization at once. Only the factor cos(*α*) of the initial magnetization is left after each measurement, where *α* is the detection flip angle. Using fixed flip angles in the experiment introduces an envelope function cos(*α*)^*i*−1^ of the observed signal, where *i* is the count of flips/radiofrequency pulses. Assuming flipping occurs every time delay TR (here 2 s), the discreet envelop function can be described by a monotonous function cos(*α*)^*t*/*TR*^ which is convenient to write in the form of e^ln(cos(*α*))*t*/*TR*^. Thus, the solution of the differential equations for the pyruvate and lactate magnetizations is as follows:4
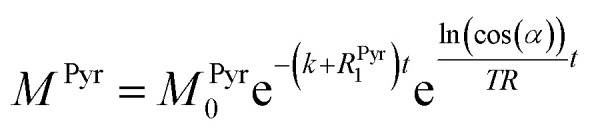
5
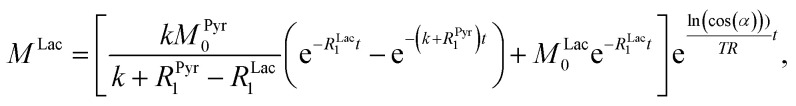
where *M*^Pyr^_0_ and *M*^Lac^_0_ are the starting magnetizations of pyruvate and lactate accordingly. Using these equations, the kinetics in Fig. 3c were fitted and the conversion rate constant *k* = 0.31 ± 0.03 s^−1^ and 0.39 ± 0.04 s^−1^ as well as the relaxation rate constant of lactate *R*^Lac^_1_ = 0.47 ± 0.03 s^−1^ and 0.50 ± 0.03 s^−1^ for two different concentrations of LDH were obtained. The relaxation rate constants of lactate should be the same in the experiments and are within the error margin. The relaxation rate at low field for the pure lactate could not be compared to a thermally polarized value due to the requirement of hyperpolarization. However, a lactate *R*^Lac^_1_ = 0.29 s^−1^ (*T*_1_ = 3.5 s) was obtained in high field at 7 T on the same sample after conversion of 2-^13^C-pyruvate-d_3_ to 2-^13^C-lactate-d_3_ (Fig. S11†) and hence is in good agreement with the relaxation rate determined at low field. The relatively short relaxation time of 2-^13^C-lactate-d_3_ may come from the influence of molecules in the final multicomponent solution that also leads to strong *T*_1_ variations in high field (Fig. S11 and S12†). Although the described technique offers many opportunities to probe a variety of biological systems, future *in vivo* applications are one option for which we discuss a few boundaries in the following. So far we have not attempted purification of the solution from catalytic impurities and other components of the reaction mixture and hence observe shorter lactate *T*_1_s as one would expect (7.2 s *T*_1_ is typical for 2-^13^C of protonated lactate).^52^ The measurement of *T*_1_ in PBS buffer that may resemble physiological conditions inside the cells where lactate is formed shows a *T*_1_ of 5.5 s (Fig. S12†). *T*_1_ of 2-^13^C-pyruvate was measured to be 42.6 s in low field (Fig. S7†) and is close to 47 s measured in high field in a murine model^53^ and close to *T*_1_ of 1-^13^C-pyruvate (∼60 s). The relaxation of 2-^13^C-pyruvate was shown to be long enough to demonstrate *in vivo* applications to brain of rats^54^ and even in humans.^53^ These are good indications that future *in vivo* studies (even in humans) are possible at such low magnetic fields and *in vivo* demonstrations will now require constructions of suitable setups. Additionally, we have recently demonstrated that our pulsed (ESOTHERIC) experiment can be performed in cost effective and portable benchtop spectrometers at 1 T to hyperpolarize metabolites and their precursors in large quantities with high efficiency.^57^ As the ESOTHERIC sequence requires weakly coupled conditions, a significant drop in polarization transfer can be expected at fields below 0.5 T. Pulsed polarization transfer experiments at millitesla fields are possible if one considers the singlet as initial state. This has already been used for several metabolites.^30,31^

With respect to the kinetic model we would like to emphasize, that the conversion rates differ by only 25% which is proportional to the LDH concentration indicating that thus determined reaction constant *k* can serve as measure of quantity of the enzyme. As a result, the presented experiments demonstrate that the real-time kinetics of enzymatic conversion can be measured and kinetic parameters determined *via* hyperpolarized NMR at very low magnetic fields. The fits for each real-time experiment are shown in the ESI† where different LDH concentrations provide different conversion rate constants.

## Experimental

Unsaturated precursor of 2-^13^C pyruvate was synthesized according to procedure described in the ESI† and in the literature. All non-synthesized chemicals were obtained from Sigma-Aldrich. Lactate dehydrogenase is from porcine heart. A solution of 1 μL of the precursor in 0.1 ml C_2_H_5_OD (44 mM) together with hydrogenation catalyst ([1,4-bis(diphenylphosphino)butane] (1,5-cyclooctadiene)rhodium(i) tetrafluoroborate) (1 mM) was placed into a 5 mm NMR tube and was hydrogenated with 83% *para*-enriched hydrogen gas (Bruker PHG 90) inside the 5 mm probehead of a 7 T Bruker (Avance III) spectrometer at 320 K. The hydrogen gas was delivered to the solution using a home-build, automated setup. Using a modified ESOTHERIC pulse sequence, the polarization was transferred from protons to the 2-^13^C-enriched pyruvate moiety. Then 0.1 ml 100 mM Na_2_CO_3_ solution in D_2_O was added to cleave the side chain and to obtain free and hyperpolarized pyruvate. The enzymatic reaction was conducted inside the low field setup after transfer of the sample. 0.3 mL 30 μL mL^−1^ LDH (94 units per experiment) or 40 μL mL^−1^ LDH (125 units per experiment), 20 mM NADH and 20% HEPES (1 M) dissolved in D_2_O was added to hyperpolarized pyruvate solution to initiate the conversion. The low field setup consists of a custom made temperature stabilized electromagnet generating a 24 mT field using a commercial power supply with 10 ppm stability. The low field spectrometer is a Magritek Kea.^2^ More experimental details and synthetic procedures can be found in the ESI.†

## Conclusions

In this work we have introduced the feasibility to perform real-time kinetic experiments of an enzymatic conversion combining hyperpolarization and NMR at very low magnetic fields (millitesla) utilizing distinctive spin–spin couplings of the metabolites. Foremost, our realization was achieved due to the possibility to obtain highly polarized 2-^13^C-pyruvate-d_3_ generated in seconds using *para*-hydrogen. Modeling the acquired data allowed us to obtain the rate constant of the enzymatic conversion from pyruvate to lactate that follows LDH concentration. This reaction is especially relevant because a prominent pyruvate–lactate metabolism is observed in cancer cells and tumors and hyperpolarized pyruvate (1-^13^C- and 2-^13^C-pyruvate) is being assessed in clinical studies for disease detection. It is worth to mention that our approach is not limited to perdeuterated pyruvate but can be applied to protonated 1-^13^C- and 2-^13^C-pyruvate by further optimizing the ESOTHERIC pulse sequence. As a consequence of our results, we believe that a series of new applications will emerge allowing for portable low field magnetic resonance to detect diseases in combination with hyperpolarization. This includes affordable low field magnetic resonance imaging (MRI) devices.^55^ Lastly, we foresee that analytical possibilities will be unlocked for ZULF-NMR in the future. This can be achieved by combining the presented method with highly sensitive detection schemes that include the EHQE (external-high-quality-enhanced) NMR approach for Faraday detection^56^ or the use of SQUIDs, atomic magnetometers and NV diamond magnetometers with the potential to measure real-time kinetics of metabolites in single cells.^32–47^

## Conflicts of interest

There are no conflicts to declare.

## Supplementary Material

SC-012-D0SC04884D-s001
