# Supplementary material for: Signal-enhanced real-time magnetic resonance of enzymatic reactions at millitesla fields
Source: Chem Sci. 2020 Oct 30;12(1):314–9. doi: 10.1039/d0sc04884d (PMC8178804; doi:10.1039/d0sc04884d)
Supplement: SC-012-D0SC04884D-s001 [file SC-012-D0SC04884D-s001.pdf]

Electronic Supplementary Material (ESI) for Chemical Science.
This journal is © The Royal Society of Chemistry 2020

Supplementary material

Signal-enhanced real-time magnetic resonance of enzymatic reactions at millitesla fields

Sergey Korchak, Anil P. Jagtap, Stefan Glöggler*

General procedure

The high field system is a Bruker ultrashield 300 MHz spectrometer and equipped with a temperature control system and a probehead with Z-gradient. Chemical shifts (δ) are given in parts per million (ppm) relative to chloroform-d1 (7.26 ppm for ^{1}H and 77.16 ppm for ^{13}C) or to water signal (4.7 ppm) in D_2O solutions.

Low field experiments were conducted with a home-built electromagnet system (24 mT) and a Kea^2 console from Magritek, see below.

All the chemicals except the synthesized compounds were purchased from Sigma Aldrich and used as received. Lactate dehydrogenase (LDH) (Sigma-Aldrich L7525), has 10544 units per ml solution, one unit will reduce 1.0 µmole of pyruvate to L-lactate per min at pH 7.5 at 37 °C.

Typical experiments are depicted in Figure S1. A solution of 1 uL of the precursor in 0.1 ml C_2H_5OD (44 mM) together with hydrogenation catalyst ([1,4-Bis(diphenylphosphino)butane] (1,5-cyclooctadiene)rhodium(I) tetrafluoroborate) (1 mM) inside a 5 mm NMR tube was hydrogenated by 83% para-enriched hydrogen gas (Bruker PHG 90) inside a probehead of a 7 T cryomagnet spectrometer at 320K. The para-enrichment was calculated from the measured amount of ortho-hydrogen in the gas phase. The parahydrogen gas was delivered to the solution by bubbling using a home-build, automated setup. The para-hydrogen gas was kept at 7 bar to achieve a higher concentration of the dissolved gas and thus to increase the rate of the hydrogenation reaction. Using a modified ESOTHERIC pulse sequence,[1-3] the 2-^{13}C-pyruvate-d$_3$ was hyperpolarized. Subsequently 0.1 ml 100 mM Na_2CO_3 solution in D_2O was added to obtain free polarized 2-^{13}C-pyruvate-d$_3$ by a base cleavage of the ester bond of the precursor. The enzymatic reaction was performed inside the cryomagnet or in the low field setup after transfer of the sample. 0.3ml of 20 µl/ml LDH (63 units per sample) (high field) or 0.3 ml of 30 µl/ml LDH (94 units per sample) and 40 µl/ml LDH (125 units per sample) (low field), 20 mM NADH and 20% HEPES buffer dissolved in D_2O was added to the hyperpolarized pyruvate solution and mixed for 2 s to initiate the conversion.

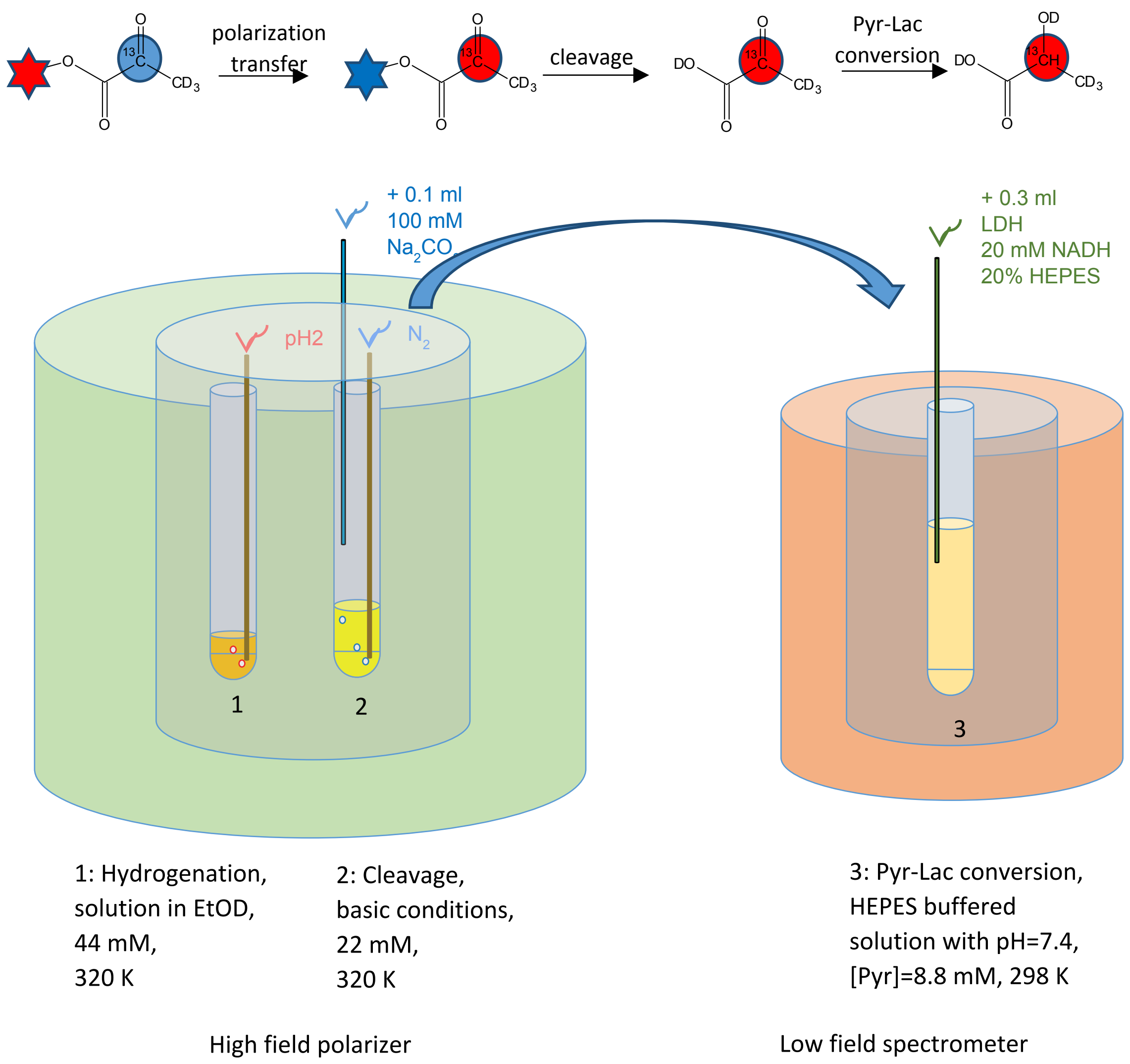

Figure S1. Scheme of experiment with hyperpolarization in high field and detection in low field.

Low field setup

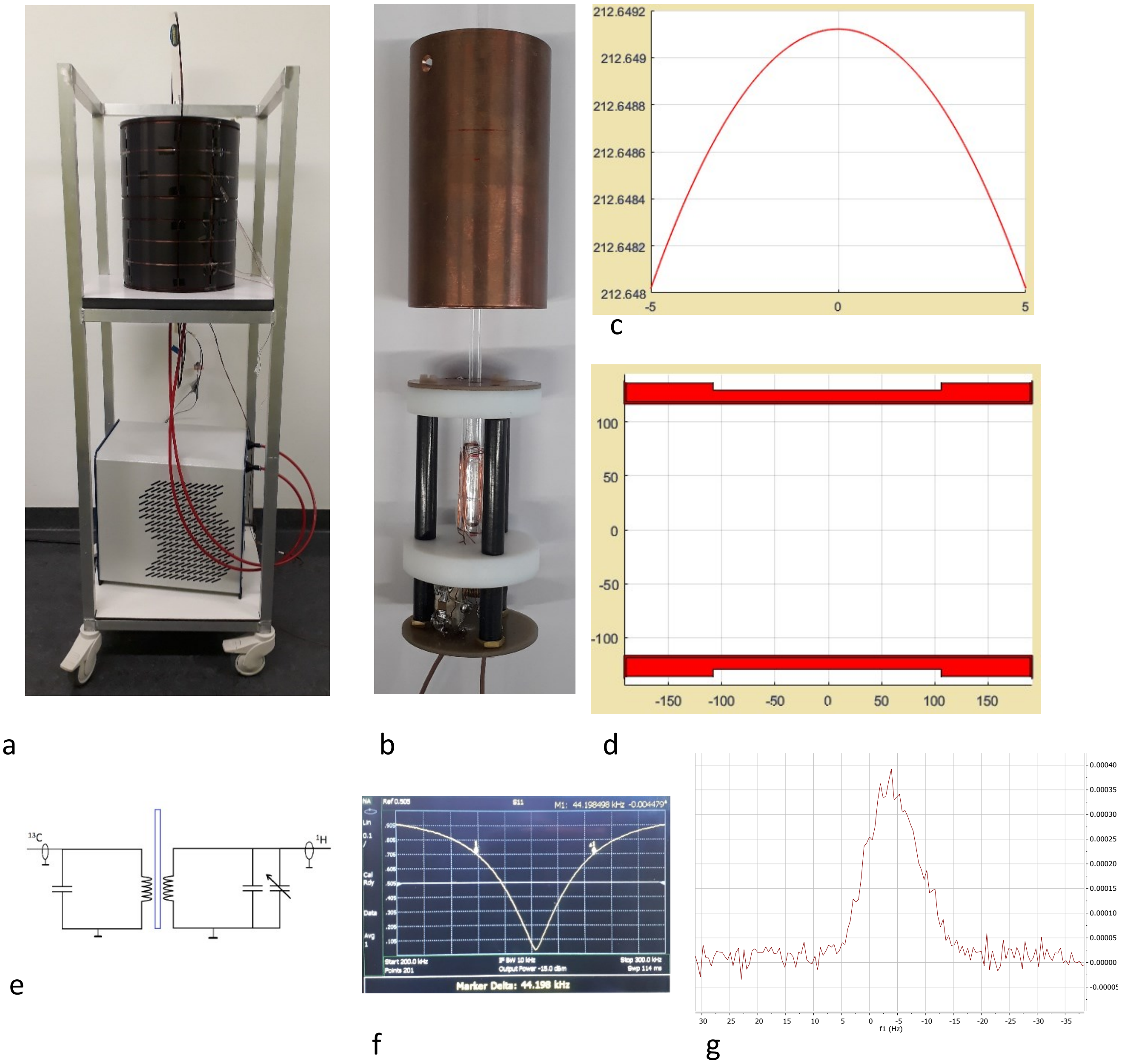

Figure S2. a) Home-built electromagnet with sets of four shims and a water cooler, b) opened probehead with inserted 5 mm NMR tube, c) simulated field profile of the electromagnet without shims in gauss (G) corresponding to 5 ppm inhomogeneity on the axis within 10 mm, d) main coil wire profile in mm: 380 mm length, 237.2 mm inner diameter, 259.2 mm first outer diameter and 272.2 mm second outer diameter, two additional wire blocks on both sides are 81.6 mm in length, e) employed circuit of two orthogonal saddle-shape B_1 coils, f) resonance profile of the inner B_1 coil with a resonance at 250 kHz, g) shimmed ^1H spectrum of water detected via the outer B_1 coil at 1 MHz, 100 averages with 1 s repetition rate.

The magnetic field for low field measurements is generated by a home-built solenoid electromagnet with sets of four shims. The main electromagnet is driven by a commercial high precision power supply (PTNhp 65-10, Heinzinger) .Two commercial power supplies (GPS-2303, Gwinstek) with four outputs are used for a shim system. The main electromagnet is wounded on an aluminum frame with 20 cm inner- and 30 cm outer-diameter using rectangular 1x4 mm wire in profile. The profile of wounded wire is shown in Fig S2d. On the outer frame grooves are made where the shim coils are wounded providing Z1, Z2, X, Y gradients. Z1 is a Helmholtz coil, Z2 is an anti-Helmholtz coil, X and Y are saddle-shape coils orthogonal to each other. The calculated field homogeneity without shims is 5 ppm on the axis over 10 mm. (Figure S2c). The design follows Ref.4 where more details on field simulations, magnet and shim designs can be found. In addition, the main aluminum frame is temperature stabilized with water from a chiller (PCMin 04.02, National Lab), to 0.1°C and usually kept at 25°C. Around 200 W heat is produced by the electromagnet. The electromagnet is powered with 8 A, producing a 24 mT field. The limitation of the field homogeneity is introduced by the main power supply with ~10 ppm specified stability. The best achieved line widths with shims are 10 Hz for ^{1}H and 4 Hz for ^{13}C for samples in 5 mm NMR tubes corresponding to 12 ppm for ^{13}C (^{13}C was measured only on hyperpolarized samples without deuterium decoupling).

A probe-head was built from a standard plastic (Figure S2b). Two transmit/receive saddle shape coils were constructed in orthogonal orientation. The inner coil with 5 turns and a 147 nF tuning capacitor was wound on a 6 mm quartz tube and used for ^{13}C (or optionally for ^{1}H) excitation and detection at ~250 kHz frequency. The outer coil with 2 turns and a 47 nF tuning capacitor was wound on a 10 mm glass tube and used for ^{1}H at 1 MHz. Both coils are ~30 mm long and orthogonal to each other and are made of 0.25 mm diameter standard copper wire with insulation. No matching capacitors were used and the impedance reached 46 Ohm.

Low field experiments were performed using a Magritek Kea^2 spectrometer. It has two channels and can be operated from 0.1-1 MHz. The internal Tx switch and power amplifier of 1 W were used to drive the B_1 coils. 1 W power was used throughout the experiments resulting in 90° pulses of 8 µs for ^{1}H and 32 µs for ^{13}C excitation. The outer coil was used only to detect the shimmed ^{1}H spectrum of water utilizing pulse of 8 µs.

Hydrogenation and polarization transfer in high field

After preparation of a solution of ethanol-OD and substrate, the pyruvate moiety reversibly forms a semiketal with ethanol. Both oxo- and semiketal-pyruvate forms participate efficiently in the hydrogenation reaction and polarization transfer due to similar *J*-couplings.

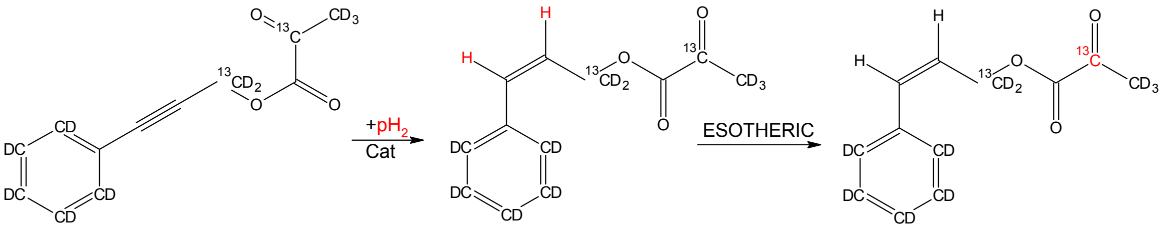

Oxo-pyruvate form

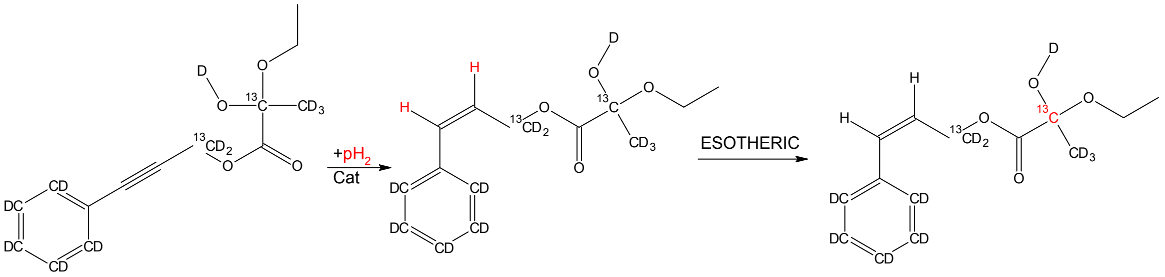

Semiketal-pyruvate form

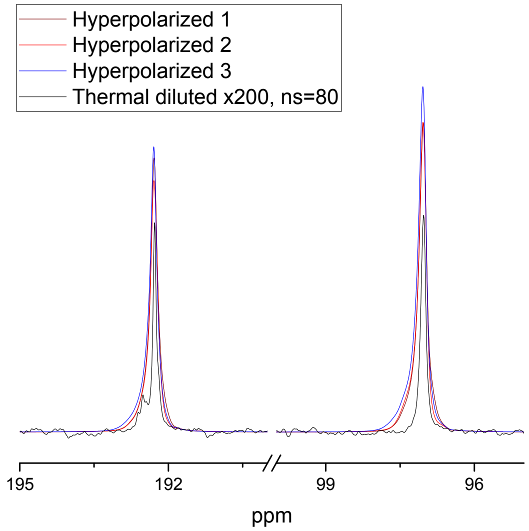

Figure S3. Top: Reaction scheme with polarization transfer of two forms of pyruvate: oxo-2-^{13}C-pyruvate-d_3 (192 ppm) and semiketal-2-^{13}C-pyruvate-d_3 (98 ppm).

Bottom: ^{13}C spectra after polarization transfer to the 2-^{13}C-pyruvate-d_3 moiety and *before* cleavage (Hyperpolarization 1,2,3). The volume of the samples are 0.2 ml. Three repeated experiments with 24.4%, 22.8%, 27.0% total polarization are calculated in comparison to an external standard of known concentration and assuming 100% hydrogenation. The hydrogenated sample were combined and diluted by C_2D_5OD 1.28 times for lock to obtain large volume of 0.4 ml. This sample (Thermal diluted) was measured to determine the concentration of the final hydrogenated products: total concentration of reaction products is 44 mM. Using this value and external standard of 0.2 ml the polarization level of hyperpolarized sample was determined.

Polarization transfer in high field

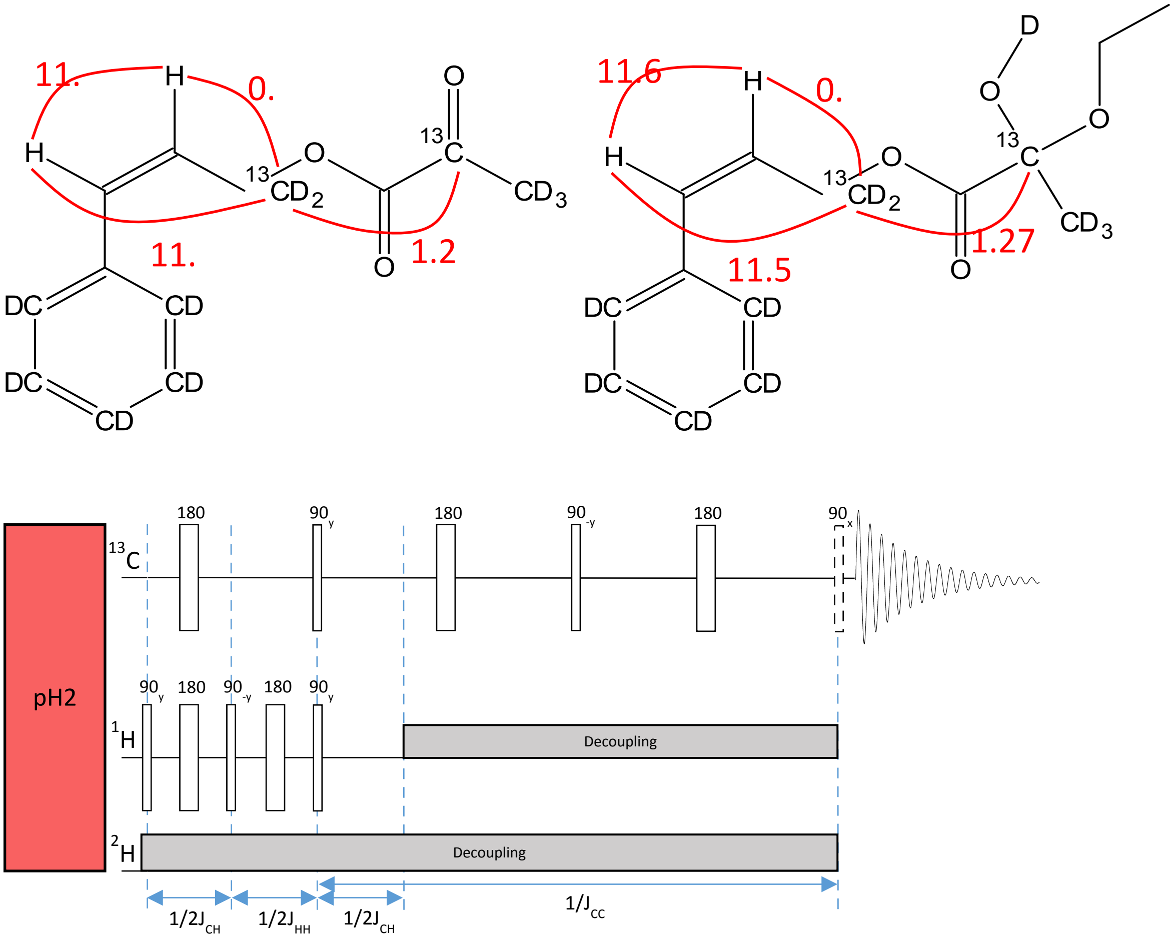

Figure S4. Top: *J*-coupling network of two structures (oxo form on the left and semiketal on the right) present after para-hydrogenation and during the polarization transfer. *J*-couplings relevant for the polarization transfer are J_{HH}=11.6 Hz, J_{CH}=11.53 Hz and J_{CC}=1.27 Hz.

Bottom: Pulse sequence for spin order transfer from para-hydrogen protons to ^{13}C of 2-^{13}C-pyruvate-d_3 in high field is the ESOTHERIC sequence with included deuterium decoupling. For decoupling the MLEV sequence was used. The last 90_x pulse on the ^{13}C channel is used to convert coherence to magnetization before hydrolysis. More details on the ESOTHERIC pulse sequence can be found elsewhere.[1-3]

Hydrolysis in high field

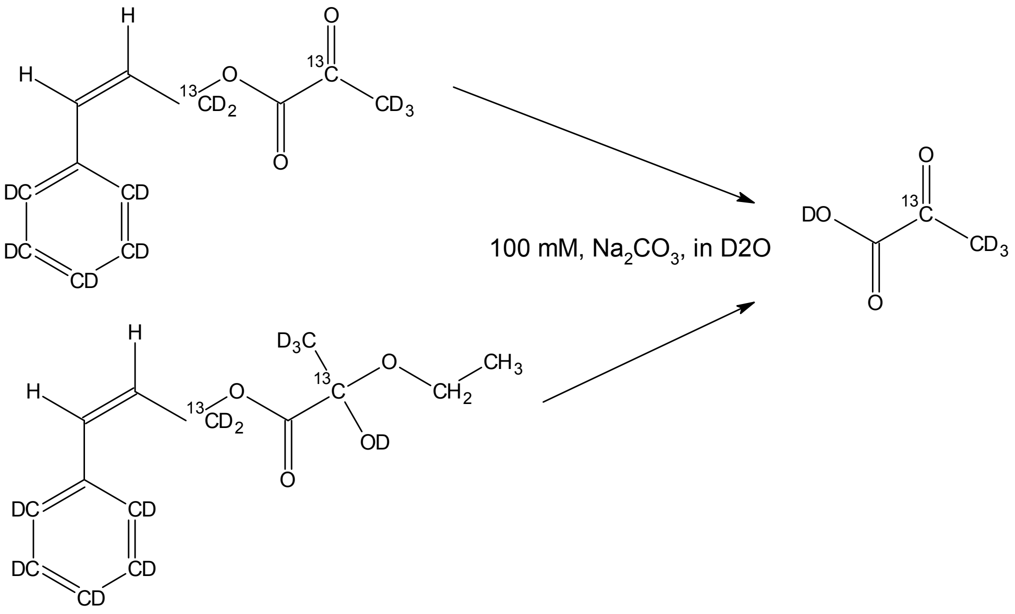

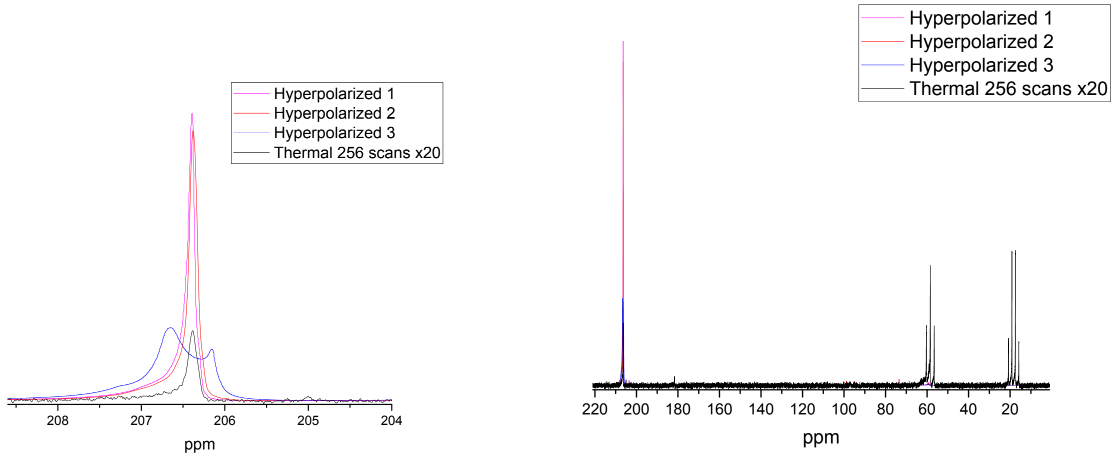

Figure S5. ^{13}C spectra with two spectral windows of thermally polarized and hyperpolarized 2-^{13}C-pyruvate-d_3 at 320 K *after* 2 s hydrolysis of 0.1 ml 44 mM hydrogenated substrate in C_2H_5OD with 0.1 ml 100 mM Na_2CO_3 in D_2O. The spectra of hyperpolarized molecules are acquired with a single 90° pulse, while the spectrum of thermally polarized molecules is a sum of 256 accumulations with 300 s repetition time and 20 times enlarged under the same conditions. The chemical shift is according to the lock on D_2O in this mixture of ethanol and water and the given temperature. Hyperpolarizations are 10.0%, 10.2%, 10.0% resulting in 10.1±0.1% on average. Only minor side products of hydrolysis is present as well as hydrated form of 2-^{13}C-pyruvate-d_3 at this basic conditions (pH~10).

Relaxation in high field after cleavage and addition of HEPES buffer

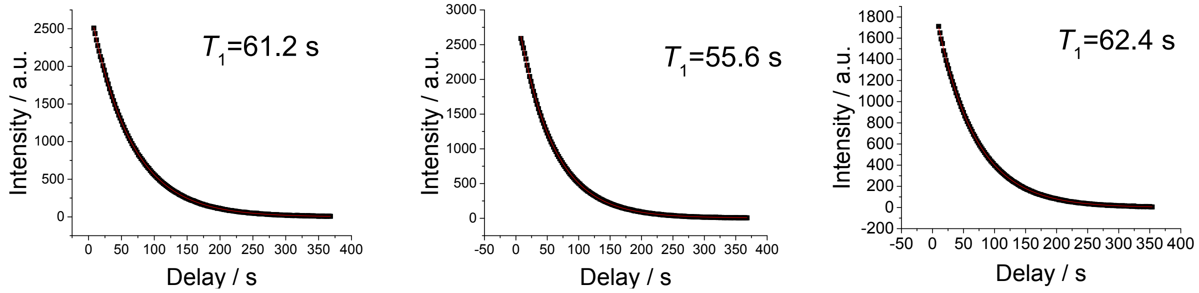

Figure S6. ^{13}C relaxation kinetics of 2-^{13}C-pyruvate-d_3 at 320 K after 2 s cleavage of 0.1 ml 44 mM hydrogenated substrate in C_2H_5OD with 0.1 ml 100 mM Na_2CO_3 in D_2O and addition of 0.3 ml 20% HEPES in D_2O. The intensities were acquired every 2 s by probing them with small flip angle (6°). The decay of the signal was corrected by dividing it with $\cos(6°)^{i-1}$, where i is the number of pulse. Three experiments give an average T_1=59.7±3.6 s.

Relaxation in low field after cleavage and addition of HEPES buffer

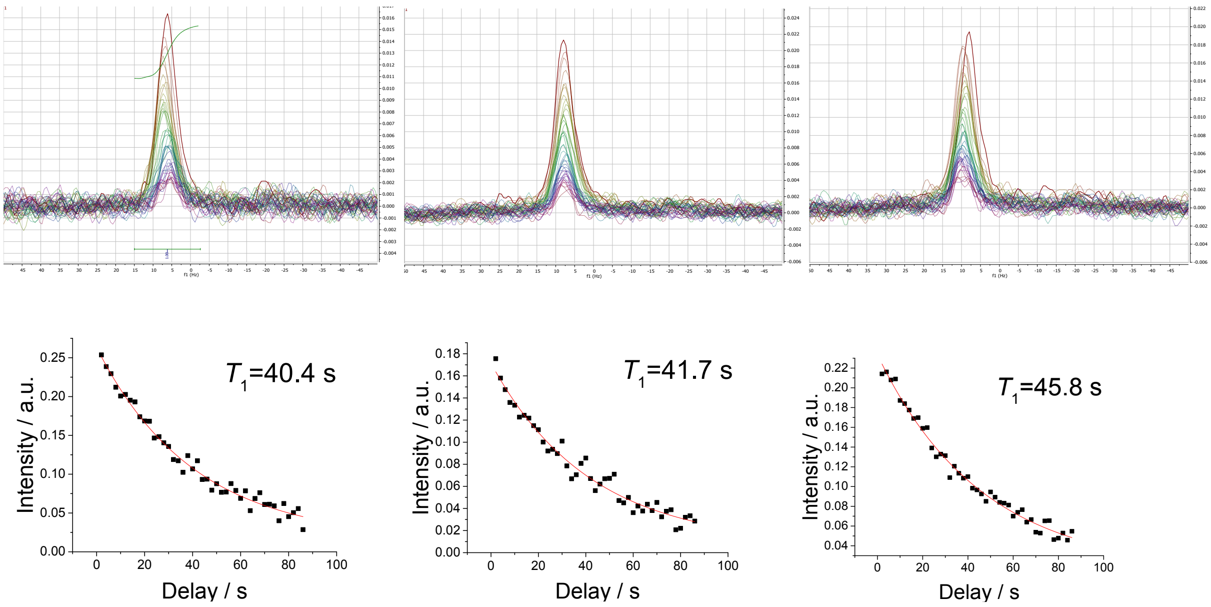

Figure S7. ^{13}C relaxation spectra with lb=1 Hz and kinetics of 2-^{13}C-pyruvate-d_3 at 298 K after 2 s cleavage of 0.1 ml 44 mM hydrogenated substrate in C_2H_5OD with 0.1 ml 100 mM Na_2CO_3 in D_2O and addition of 0.3 ml 20% HEPES in D_2O. The intensities were acquired every 2 s by probing them with small flip angle (6°) in the low field setup. The decay of the signal caused by detection was corrected by dividing it with $\cos(6°)^{i-1}$, where i is the number of pulses. Three experiments give an average T_1=42.6±2.8 s.

Real-time kinetics of enzyme pyruvate-lactate conversion in low field

The fitting of the each kinetics was done following. Firstly, each pyruvate decay was fit to the equation (2) derived in the main text:

$$M^{Pyr} = M_0^{Pyr} e^{-\left(k + R_1^{Pyr}\right)t} e^{\frac{\ln(\cos(\alpha))}{TR}t}$$

, where cos(α), TR and R_1^{Pyr} are known and fixed parameters while M_0^{Pyr} and k are fit to the curve.

Secondly, the lactate kinetics was fit to the equation (3):

$$M^{Lac} = \left[\frac{kM_0^{Pyr}}{k + R_1^{Pyr} - R_1^{Lac}}\left(e^{-R_1^{Lac}t} - e^{-(k + R_1^{Pyr})t}\right) + M_0^{Lac} e^{-R_1^{Lac}t}\right] e^{\frac{\ln(\cos(\alpha))}{TR}t}$$

, where cos(α), TR and R_1^{Pyr} are known and M_0^{Pyr} and k are found from previous fitting. M_0^{Lac} and R_1^{Lac} are fit to the curve.

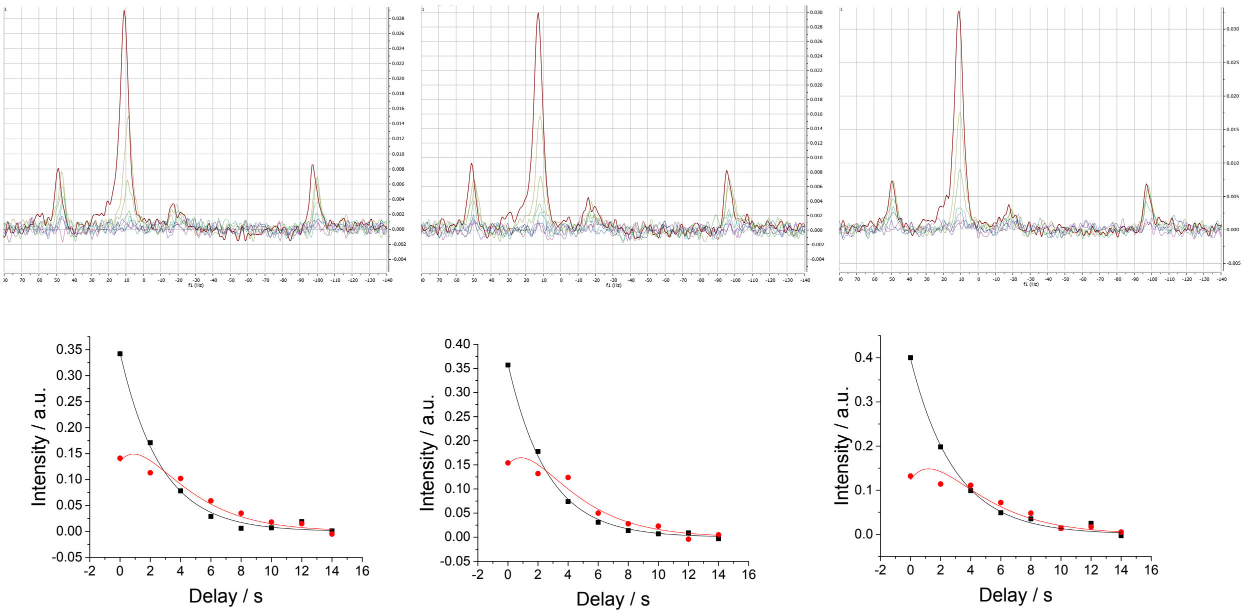

k=0.307±0.019 s^{-1}, R_1^{Lac} =0.469±0.038 s^{-1} / k=0.314±0.012 s^{-1}, R_1^{Lac} =0.456±0.038 s^{-1} / k=0.275±0.014 s^{-1}, R_1^{Lac} =0.452±0.040 s^{-1}

Figure S8. Three experiments of kinetics of enzymatic pyruvate-lactate conversion with 94 units of LDH in low field at 298 K with corresponding kinetic parameters under them.

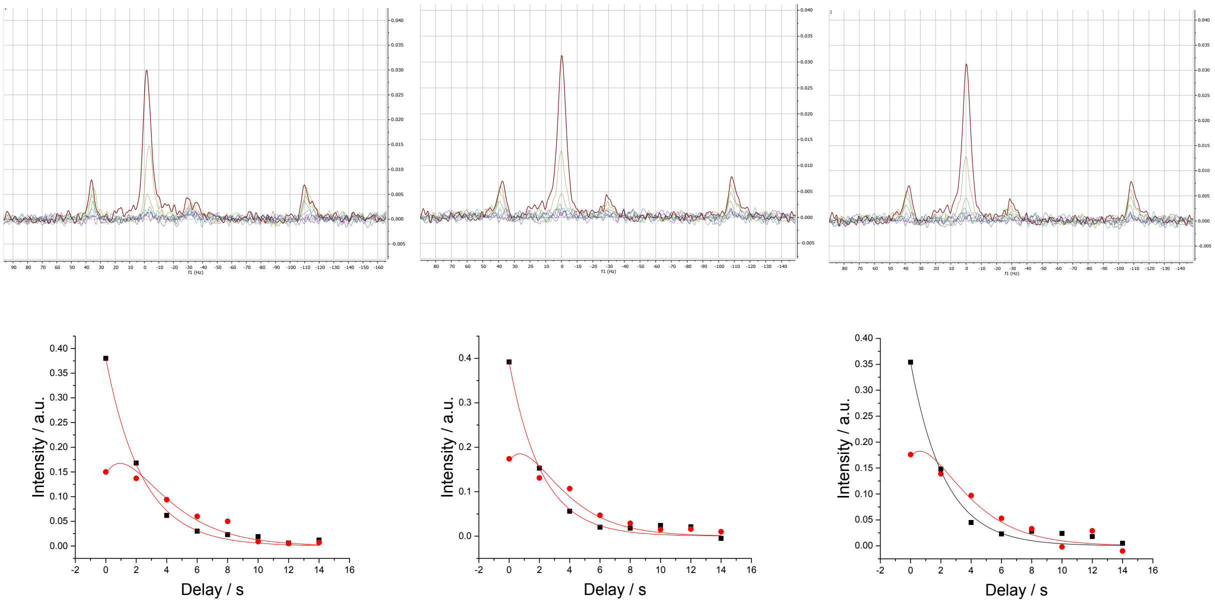

k=0.351±0.020 s^{-1}, R_1^{Lac} =0.500±0.037 s^{-1} / k=0.402±0.032 s^{-1}, R_1^{Lac} =0.570±0.051 s^{-1} / k=0.381±0.038 s^{-1}, R_1^{Lac} =0.522±0.046 s^{-1}

Figure S9. Three experiments of kinetics of enzymatic 2-^{13}C-pyruvate-d$_3$-2-^{13}C-lactate-d$_3$ conversion with 125 units of LDH in low field at 298 K with corresponding kinetic parameters under them.

Real-time kinetics of enzymatic pyruvate-lactate conversion in high field

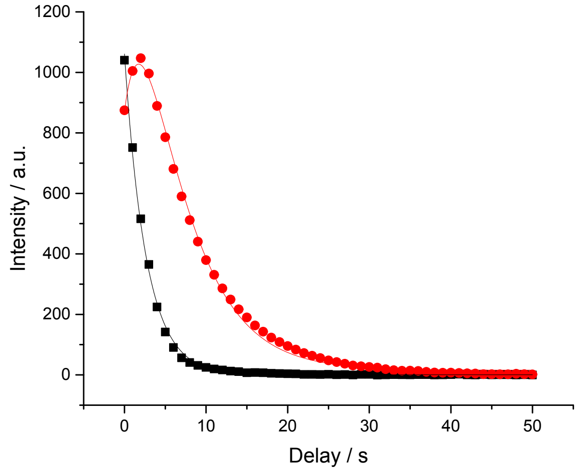

Figure S10. Kinetics of enzymatic 2-^{13}C-pyruvate-d$_3$ to 2-^{13}C-lactate-d$_3$ conversion with 63 units of LDH in high field using 6° flip angle in 1 s steps. T_1^{Pyr}=59.7±3.6 s of 2-^{13}C-pyruvate-d$_3$ was measured separately from decay of hyperpolarized signal with small flip angle. Rates obtained from fitting to the model are k=0.358±0.005 s^{-1}, R_1^{Lac} =0.194±0.002 s^{-1}. The ration rate under these conditions is higher than in low field that could be because of higher than room temperature. The relaxation time (5.2 s) correspond to the same time at lower temperature of 310 K (Figure S11) but not at 320 K (6.5 s). This can be explained by the fact that the pyruvate-lactate conversion is initiated by injecting room temperature Na_2CO_3 and LDH/NADH solutions into 320 K initial C_2H_5OD solution. Thus for several seconds the temperature of the sample is below original 320 K before spectrometer temperature control does not stabilize it back to its initial conditions.

Relaxation of 2-^{13}C-lactate-d$_3$ in high field after conversion reaction

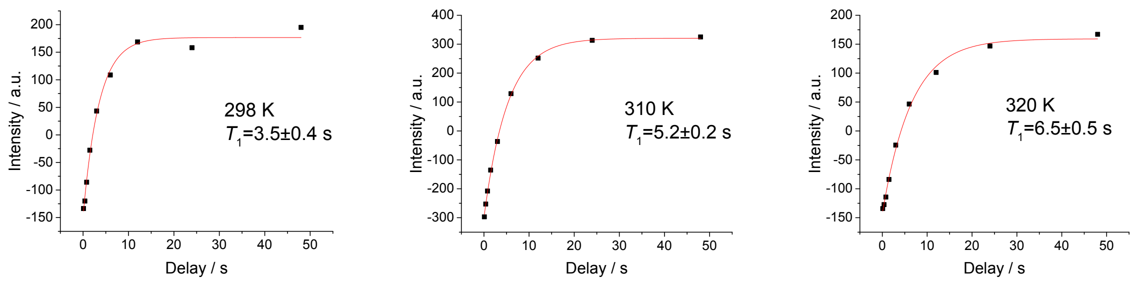

Figure S11. ^{13}C relaxation times of 2-^{13}C-lactate-d$_3$ at three temperatures 298 K, 310 K and 320 K in the solution after pyruvate-lactate conversion experiment in Figure S9 measured by inversion recovery of thermally polarized sample. The 2-^{13}C-lactate-d$_3$ was generated after cleavage of 0.1 ml 44 mM hydrogenated substrate in C_2H_5OD with 0.1 ml 100 mM Na_2CO_3 in D_2O and conversion to 2-^{13}C-lactate-d$_3$ by addition of 0.3 ml 20% HEPES in D_2O with 125 units LDH (40ul/ml). 8 scans were accumulated for each point.

Relaxation of non-labelled lactate in high field after the conversion reaction

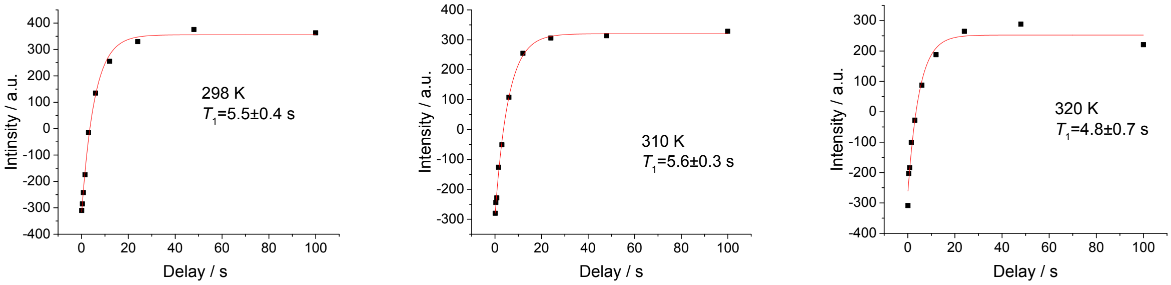

Figure S12. 2-^{13}C relaxation times of 125 mM non-labelled lactic acid at three temperatures in PBS buffer (H_2O) with 10% D_2O measured (pH=7.2) by inversion recovery of thermally polarized sample. 100 scans were accumulated for each point.

Synthesis of side-arm hydrogenative derivative-pyruvate ester

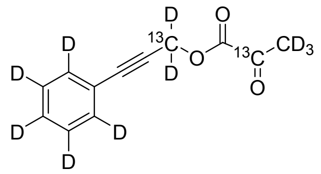

Abbreviations

CuI	Copper(I) iodide
CH_2Cl_2	Dichloromethane
d	Doublet
DCl	Deuterium chloride
DMF	*N*,*N*-Dimethylformamide
DMSO	Dimethyl sulfoxide
EtOAc	Ethyl acetate
Et_3N	Triethylamine
HCl	Hydrochloric acid
KOH	Potassium hydroxide
Pd $(PPh_3)_4$	Tetrakis(triphenylphosphine)palladium(0)
quint	Quintet
RBF	Round-bottom flask
t	Triplet
TBAF	Tetrabutylammonium fluoride
THF	Tetrahydrofuran
TLC	Thin layer chromatography
TMSA	Trimethylsilylacetylene

Synthesis of 2-^{13}C-sodium pyruvate-d_3 (1)

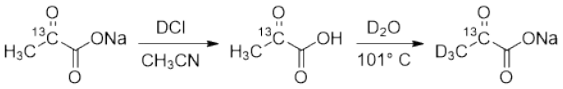

1

Acidification and deuteration

To an ice-cold solution of sodium pyruvate-2-^{13}C (1 g, 9 mmol) in 4 mL acetonitrile, DCl (0.6 mL, 38% solution in D_2O) was added dropwise. The resulting solution was further stirred at the same temperature for another 30 min. The solid obtained (Sodium chloride) during the neutralization process was filtered through a Buchner funnel. The filtrate obtained was concentrated under reduced pressure resulting in yellowish colored liquid. To this residue acetone (4 mL) and Na_2SO_4 (0.3 g) were added and kept it the freezer for 30 min. Small traces of solids obtained were again filtered through a Buchner funnel. The filtrate obtained was concentrated under vacuum to get yellowish oily material (0.8 g, 90%). Deuteration of this material was carried out according to the reported procedure.[5]

Synthesis of 13C-propargyl bromide derivative (5)

Synthesis of ^{13}C-propargyl bromide (**5**) was done as following:

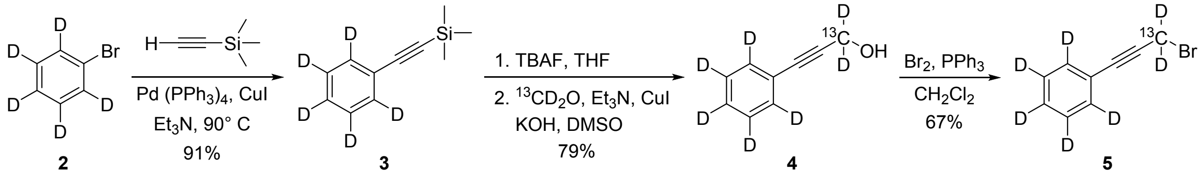

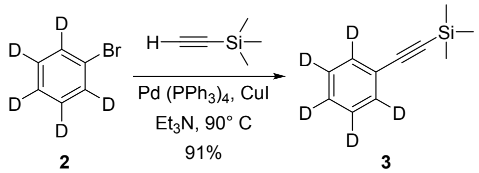

Sonogashira coupled product (3)

$Pd(PPh_3)_4$ (0.071 g, 0.0617 mmol) and CuI (0.013 g, 0.1234 mmol) were degassed in a flame-dried RBF, which was followed by the addition of **2** (2 g, 1.29 mL, 12.3 mmol) in Et_3N (15 mL). The resulting solution was degassed again and then (trimethylsilyl)acetylene (TMSA, 1.818 g, 2.56 mL, 18.5 mmol) was added. The reaction mixture was heated at 90 °C for 24 h. A black solution was obtained, which was diluted with CH_2Cl_2 and washed with 0.1 N aq. HCl (3 x 20 mL). The aqueous solution was extracted with CH_2Cl_2 (3 x 20 mL). The combined organic layer was dried over Na_2SO_4, filtered and concentrated *in vacuo* to get crude material, which was purified by flash column chromatography (silica) with elution of pet. ether to give **3** (2.017 g, 91% yield) as a yellow liquid.

TLC (Silica gel, 100% pet. ether), R_f (**3**) = 0.4, UV active.
^1H NMR ($CDCl_3$, 298K, 300.13 MHz) δ = 0.23 (S, 6H), 0.17 (S, 3H) ppm.
^2H NMR ($CDCl_3$, 298 K, 46.1 *MHz*) δ = 7.55 (4 x ^2H), 6.86 (1 x ^2H) ppm.
^{13}C NMR ($CDCl_3$, 298K, 75.5 MHz) δ = 131.56 (t, ^{13}C-^2H, $^1J_{C,D}$ = 24.78 Hz), 127.96 (t, ^{13}C-^2H, $^1J_{C,D}$ = 24.74 Hz), 127.69 (t, ^{13}C-^2H, $^1J_{C,D}$ = 24.54 Hz), 122.89 (s), 105.08 (s), 94.10 (s),
-0.07 ppm

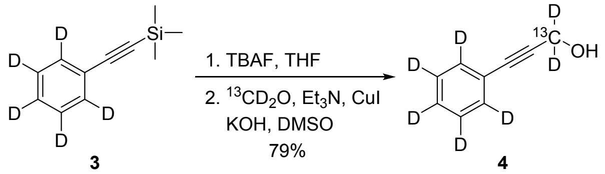

^{13}C-Propargyl alcohol derivative (4)

Deprotection of TMS group

To a cold solution of **3** (3.3 g, 18.4 mmol) in diethyl ether (15 mL), was added TBAF (24 mL, 23.9 mmol, 1 M in THF solution) dropwise over the period of 15 min. Black precipitation was observed during the addition process. The resulting solution was stirred at 27 °C for 30 min. The solution was diluted with diethyl ether (20 mL) and washed with water (30 mL). If required, it was filtered through celite bed. The organic layer was dried over Na_2SO_4, filtered and concentrated under vacuum to obtain a yellowish oily material (phenyl acetylene), which was taken ahead for the next reaction without further purification. TLC (Silica gel, 100% pet. ether), R_f (**3**) = 0.4, R_f (phenyl acetylene) = 0.5, $KMnO_4$ active.

Coupling of phenyl acetylene with $^{13}CD_2O$ (labelled formaldehyde)

To a stirred solution of above phenyl acetylene (2.7 g, 25.2 mmol) in DMSO (35 mL) was added, Et_3N (2.54 g, 8.32 mL, 25.2 mmol), CuI (0.137 g, 12.6 mmol) and KOH (1.41 g, 25.2 mmol). To this solution, labelled formaldehyde-$^{13}CD_2O$ (1.66 g, 8.32 ml, 50.4 mmol, 20% solution in D_2O) was added dropwise. The resulting solution was heated to 55 °C for 20 min. This reaction was performed in a RBF which is fitted with a condenser and open to air. After completion of the reaction, it was cooled to room temperature and was diluted with EtOAc. The organic layer was washed with water, brine, dried over Na_2SO_4 and was concentrated under reduced pressure. The crude product was purified by flash column chromatography (silica) using a gradient elution (EtOAc: pet ether; 0:100 to 10:90) to give **4** (2.034 g, 79% yield) as a yellowish liquid.

TLC (Silica gel, 100% pet. ether), R_f (terminal alkyne) = 0.5, $KMnO_4$ active.
(Silica gel, 10% EtOAc in pet. ether), R_f (**4**) = 0.3, $KMnO_4$ and UV active.

^{1}H NMR ($CDCl_3$, 298K, 300.13 MHz) δ = 1.82 (S, 1H, -OH) ppm.

^{2}H NMR ($CDCl_3$, 298 K, 46.1 MHz) δ = 7.60-6.90 (5 x ^{2}H), 4.72 (1 x ^{2}H), 4.23 (1 x ^{2}H), ppm.

^{13}C NMR ($CDCl_3$, 298K, 75.5 MHz) δ = 131.27 (t, ^{13}C-^{2}H, $^{1}J_{C,D}$ = 24.99 Hz), 127.98 (t, ^{13}C-^{2}H, $^{1}J_{C,D}$ = 24.23 Hz), 127.80 (t, ^{13}C-^{2}H, $^{1}J_{C,D}$ = 24.48 Hz), 122.29 (s), 87.64 (d, ^{13}C-^{13}C, $^{1}J_{C,C}$ = 73.64 Hz), 85.69 (d, ^{13}C-^{13}C, $^{2}J_{C,C}$ = 12.75 Hz), 51.09 (quint, ^{13}C-^{2}H, $^{1}J_{C,D}$ = 22.47 Hz) ppm

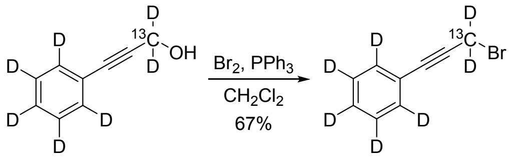

4 5

^{13}C-Propargyl bromide derivative (5)

To a solution of Ph_3P (1.234 g, 4.7 mmol) in CH_2Cl_2 (15 mL) at 0 °C under air, was added Br_2 (0.745 g, 0.240 mL, 4.66 mmol) in CH_2Cl_2 (10 mL) dropwise. The solution was stirred at 0 °C for 30 min, turning into a yellow slurry. A solution of **4** (0.6 g, 4.28 mmol) in CH_2Cl_2 (5 mL) was added dropwise. The resulting yellow solution was further stirred at 0 °C for 1 h, and then at 27 °C for 17 h. After completion of reaction, the solvent was removed *in vacuo* to get crude material which was purified by flash column chromatography on silica gel using gradient elution (EtOAc: pet ether; 0:100 to 2:98) to give **5** (0.583 g, 67% yield) as a colourless liquid.

TLC (Silica gel, 10% EtOAc in pet. ether), R_f (**4**) = 0.3, R_f (**5**) = 0.9, UV active.
^{2}H NMR ($CDCl_3$, 298 K, 46.1 *MHz*) δ = 7.62-6.92 (5 x ^{2}H), 4.44 (1 x ^{2}H), 3.9 (1 x ^{2}H) ppm.
^{13}C NMR ($CDCl_3$, 298K, 75.5 *MHz*) = 131.47 (t, ^{13}C-^{2}H, $^1J_{C,D}$ = 24.74 Hz), 128.35 (t, ^{13}C-^{2}H, $^1J_{C,D}$ = 24.09 Hz), 127.81 (t, ^{13}C-^{2}H, $^1J_{C,D}$ = 24.33 Hz), 121.90 (s), 86.77 (d, ^{13}C-^{13}C, $^2J_{C,C}$ = 13.57 Hz), 84.65 (d, ^{13}C-^{13}C, $^1J_{C,C}$ = 79.97 Hz), 15.02 (quint, ^{13}C-^{2}H, $^1J_{C,D}$ = 24.34 Hz) ppm.

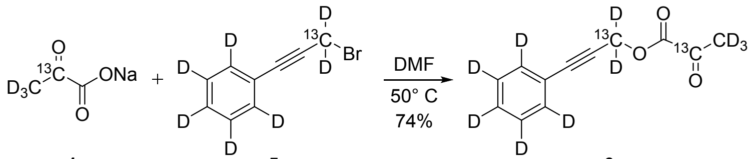

1 5 6

Synthesis of ^{13}C-pyruvate ester (6)

To a solution of **5** (0.300 g, 2.63 mmol) in DMF (10 mL), was added **1** (0.333 g, 1.64 mmol). The resulting suspension was heated at 50 °C for 7 h. After completion reaction, it was cooled to 0 °C and quenched by adding water to it (25 mL). To this solution, EtOAc (25 ml) was added. The organic layer was separated and the aqueous layer was again extracted with EtOAc (15 ml). The combined organic layer was washed with water, brine, dried over Na_2SO_4 and was concentrated under reduced pressure. The crude product was purified by flash column chromatography (silica) using a gradient elution (CH_2Cl_2) to give **6** (0.260 g, 74% yield) as a colorless liquid.

TLC (Silica gel, 10% EtOAc in pet. ether), R_f (**5**) = 0.9, R_f (**6**) = 0.3, UV active.
^{2}H NMR ($CDCl_3$, 298 K, 46.1 *MHz*) δ = 7.56-7.32 (5 x ^{2}H), 5.26 (1 x ^{2}H), 4.75 (1 x ^{2}H), 2.50-2.433 (3 x ^{2}H) ppm.

^{13}C NMR ($CDCl_3$, 298K, 75.5 *MHz*) = 191.01 (d, ^{13}C-^{13}C, $^3J_{C,C}$ = 1.36 Hz), 160.33 (dd, ^{13}C-^{13}C, $^1J_{C,C}$ = 67.03 and $^2J_{C,C}$ = 2.9 Hz), 131.53 (t, ^{13}C-^{2}H, $^1J_{C,D}$ = 24.99 Hz), 128.53 (t, ^{13}C-^{2}H, $^1J_{C,D}$ = 23.88 Hz), 127.83 (t, ^{13}C-^{2}H, $^1J_{C,D}$ = 24.66 Hz), 121.49 (s), 87.65 (d, ^{13}C-^{13}C, $^2J_{C,C}$ = 13.86 Hz), 81.90 (d, ^{13}C-^{13}C, $^1J_{C,C}$ = 81.43 Hz), 54.07 (quint, ^{13}C-^{2}H, $^1J_{C,D}$ = 23.65 Hz), 26.48 (m, broad) ppm.

HRMS: calculated for $C_{10}{}^{13}C_2D_{10}O_3Na^+$ $[M+Na]^+$ 237.13, found 237.20

References:

1 S. Korchak, M. Emondts, S. Mamone, B. Blümich and S. Glöggler, *PCCP*, 2019, **21**, 22849-22856.

2 S. Korchak, S. Mamone and S. Glöggler, *ChemistryOpen*, 2018, **7**, 672-676.

3 S. Korchak, S. Yang, S. Mamone and S. Glöggler, *ChemistryOpen*, 2018, **7**, 344-348.

4 J. F. P. Colell, PhD thesis, RWTH Aachen University, 2015.

5 R. V. Shchepin, A. M. Coffey, K. W. Waddell and E. Y. Chekmenev, *Analytical chemistry*, 2014, **86**, 5601-5605.
